# Antibiotics and the Human Gut Microbiome: Dysbioses and Accumulation of Resistances

**DOI:** 10.3389/fmicb.2015.01543

**Published:** 2016-01-12

**Authors:** M. P. Francino

**Affiliations:** ^1^Unitat Mixta d’Investigació en Genòmica i Salut, Fundación para el Fomento de la Investigación Sanitaria y Biomédica de la Comunitat Valenciana (FISABIO)-Salud Pública/Institut Cavanilles de Biodiversitat i Biologia Evolutiva, Universitat de ValènciaValència, Spain; ^2^Consorcio de Investigación Biomédica en Red de Epidemiología y Salud PúblicaMadrid, Spain

**Keywords:** antibiotics, human gut microbiota, autoimmunity, immunotolerance, atopy, inflammation, dysbiosis, resistance reservoir

## Abstract

The human microbiome is overly exposed to antibiotics, due, not only to their medical use, but also to their utilization in farm animals and crops. Microbiome composition can be rapidly altered by exposure to antibiotics, with potential immediate effects on health, for instance through the selection of resistant opportunistic pathogens that can cause acute disease. Microbiome alterations induced by antibiotics can also indirectly affect health in the long-term. The mutualistic microbes in the human body interact with many physiological processes, and participate in the regulation of immune and metabolic homeostasis. Therefore, antibiotic exposure can alter many basic physiological equilibria, promoting long-term disease. In addition, excessive antibiotic use fosters bacterial resistance, and the overly exposed human microbiome has become a significant reservoir of resistance genes, contributing to the increasing difficulty in controlling bacterial infections. Here, the complex relationships between antibiotics and the human microbiome are reviewed, with focus on the intestinal microbiota, addressing (1) the effects of antibiotic use on the composition and function of the gut microbiota, (2) the impact of antibiotic-induced microbiota alterations on immunity, metabolism, and health, and (3) the role of the gut microbiota as a reservoir of antibiotic resistances.

## Effects of Antibiotics on the Gut Microbiota

Several lines of evidence confirm that antibiotic administration can result in gut microbiota dysbiosis, i.e., disturbance in composition and function. Broad-spectrum antibiotics can affect the abundances of 30% of the bacteria in the gut community, causing rapid and significant drops in taxonomic richness, diversity and evenness (Dethlefsen et al., 2008; Dethlefsen and Relman, 2011). Once antibiotic treatment has stopped, the microbiota may present a certain degree of resilience, being capable of returning to a composition similar to the original one, but the initial state is often not totally recovered. In fact, antibiotic-induced microbiota alterations can remain after long periods of time, spanning months and even years (De La Cochetiere et al., 2005; Jernberg et al., 2007; Dethlefsen et al., 2008; Dethlefsen and Relman, 2011). In infants, few studies have gaged the extent to which gut microbiota development is affected by early exposure to antibiotics, in spite of the major impact of this process on life-long health. Tanaka et al. (2009) and Fouhy et al. (2012) studied the microbiota of infants treated with antibiotics in the first days of life, and reported effects within 1 week and within 2 months after birth. Early antibiotic exposure both reduced the diversity of the infants’ microbiota and altered its composition, with an attenuation of *Bifidobacterium* and marked increases of Proteobacteria. Moreover, the microbiota of those infants who were not treated, but whose mothers received antibiotics prior to delivery, showed the same alterations seen in the microbiota of the treated infants (Tanaka et al., 2009).

The impact of antibiotics on the gut microbiota has more recently been investigated through the variety of “omic” techniques available today for microbial community analyses (reviewed in Franzosa et al., 2015). These works have shown that, beyond altering the composition of taxa, antibiotics also affect the gene expression, protein activity and overall metabolism of the gut microbiota. These changes can occur at a much faster pace than those involving replacement of taxa in the community (Perez-Cobas et al., 2012). Moreover, the induced changes can drive the functionality of the microbiota toward states similar to those observed under disease conditions. In this direction, the microbiota of individuals treated with β-lactams has a repertoire of enzymatic activities for carbohydrate degradation that results in an unbalanced sugar metabolism, similar to that observed in obese individuals (Hernandez et al., 2013). Experimental approaches have also confirmed that antibiotics rapidly alter the physiological state and activity of the gut microbiota. In *ex vivo* incubations of fecal samples with different antibiotics, there was an increase in the proportion of gut microbiota cells with damaged membranes, the active populations of the microbiota changed, and genes involved in antibiotic resistance, stress response and phage induction augmented in expression (Maurice et al., 2013). In addition, expression also increased for genes related to genetic information processing (e.g., transcription and translation) in the case of antibiotics that inhibit translation, such as tetracycline and the macrolides. The substantial effects documented for antibiotics on the functioning of the gut microbiota stress the likely impact that antibiotic exposure will have on the physiological processes that depend on the activities performed by the microbes in this community.

## Effects of Antibiotic-Induced Microbiota Alterations on Immune and Metabolic Health

### Increased Susceptibility to Infections

One of the most imminent threats of gut microbiota alterations is the increased susceptibility to intestinal infections, which can stem from newly acquired pathogens or from the sudden overgrowth and pathogenic behavior of opportunistic organisms already present in the microbiota. In particular, antibiotic-associated diarrheas (AAD) due to nosocomial pathogens are a frequent occurrence. These are often associated with organisms such as *Klebsiella pneumoniae*, *Staphylococcus aureus* and, of most concern, *Clostridium difficile*, which can cause intractable, long-term recurrent infections and even a potentially lethal pseudomembranous colitis (Wilcox, 2003; Young and Schmidt, 2004; Song et al., 2008; Rupnik et al., 2009; Sekirov et al., 2010; Chen et al., 2013). A mouse model has provided evidence that the substantial losses of diversity caused by antibiotics in the small and large intestinal microbiota can result in the establishment of a chronic infection with *C. difficile* (Lawley et al., 2009; Buffie et al., 2012).

In addition, bloodstream infection in immunocompromised individuals is another life-threatening condition that increases in risk due to antibiotic treatment. In the clinical setting, intestinal domination by vancomycin-resistant *Enterococcus* has been shown to precede bloodstream infection by this pathogen, and experimental work in mice has established that antibiotic treatment sets the stage for the intestinal outgrowth of this bacterium (Ubeda et al., 2010). In premature infants, who are heavily treated with broad-spectrum antibiotics, the risk of sepsis has also been related to gut microbiota composition and length of antibiotic treatment (Madan et al., 2012; Mai et al., 2013).

### Compromised Immune Homeostasis and Tolerance

The microbiota alterations caused by antibiotics, beyond increasing the immediate risk for infection, can also affect basic immune homeostasis with body-wide and long-term repercussions. Atopic, inflammatory and autoimmune diseases have been linked to gut microbiota dysbiosis, and, in some cases, significant associations have been established between these diseases and the intake of antibiotics during early life. Clearly, the effects of antibiotic-induced dysbiosis will be even more relevant if they occur early in life, a critical period for maturation of the immune system and establishment of immunological tolerance (Francino, 2014).

In the case of atopic diseases, numerous studies have demonstrated links to the composition of the gut microbiota during infancy and early childhood (Kuvaeva et al., 1984; Wold, 1998; Penders et al., 2006; Wang et al., 2008; Bisgaard et al., 2011; Abrahamsson et al., 2012). Most recurrently, a significant association has been detected with bifidobacteria deficiency (Sepp et al., 1997, 2005; Bjorksten et al., 1999; Kalliomaki et al., 2001; Mah et al., 2006; Sjogren et al., 2009). However, this link could not be upheld in two large prospective case-control studies (Murray et al., 2005; Penders et al., 2006). As not all *Bifidobacterium* species appear to have a protective role (He et al., 2001; Ouwehand et al., 2001; Sjogren et al., 2009), these discrepancies among studies could be due to the presence of different bifidobacteria in different geographical regions, in addition to the likely contribution of genetic variation among human populations. On the other hand, high abundances of organisms such as *Clostridium coccoides* and *Escherichia coli* and a microbiota of low diversity have also been repeatedly associated to the presence of different atopic diseases (Bjorksten et al., 2001; Sepp et al., 2005; Mah et al., 2006; Wang et al., 2008; Bisgaard et al., 2011; Thompson-Chagoyan et al., 2011; Abrahamsson et al., 2012). These associations suggest that early antibiotic use would likely increase the risk for atopic disease, but the existence of such link has been controversial (Bedford Russell and Murch, 2006; Kuo et al., 2013). Retrospective epidemiological studies have generally supported the association (Alm et al., 1999; von Mutius et al., 1999; Wickens et al., 1999; Droste et al., 2000; Wjst et al., 2001; Thomas et al., 2006; Foliaki et al., 2009), but most prospective analyses have failed to do so (Illi et al., 2001; Celedon et al., 2002, 2004; Harris et al., 2007; Wickens et al., 2008; Mai et al., 2010; Su et al., 2010). Nevertheless, the application of techniques aimed at reducing potential biases and confounding effects has resulted in the detection of dose-dependent associations between asthma and early life exposure to antibiotics in several prospective studies (McKeever et al., 2002; Kozyrskyj et al., 2007; Marra et al., 2009). Moreover, broad-spectrum antibiotics show a stronger association with asthma, indicating that a reduction of bacterial diversity in the microbiota is likely to contribute to the effect of antibiotics on asthma development (McKeever et al., 2002; Kozyrskyj et al., 2007). In addition to asthma, other allergic outcomes have also recently been associated with early intake of antibiotics (Risnes et al., 2011). Similarly, the risk for several atopic diseases is increased by maternal intake of antibiotics during pregnancy, in a dose-dependent manner (Jedrychowski et al., 2006).

Gut microbiota composition has also been linked to numerous disorders involving processes of inflammation and autoimmunity. This is the case of necrotizing enterocolitis (NEC), a devastating inflammatory disease for newborns. A low abundance of *Bifidobacterium*, accompanied by a generally low bacterial diversity, has been detected before NEC onset (Mai et al., 2011, 2013). Morover, populations exposed to antibiotics, such as preterm infants (Deshpande et al., 2010) and infants whose mothers receive antibiotics in order to defer labor (Kenyon et al., 2001), present an increased incidence of NEC. Chron’s disease (CD), another inflammatory bowel disease (IBD), also increases in those children who receive antibiotics before 5 years of age (Hildebrand et al., 2008). This disease was one of the first for which an association with the human gut microbiota was clearly established through metagenomic analyses, with a reduction in Firmicutes (particularly *C. leptum*) and an increase of some Gram-negative bacteria (Porfiromonadaceae) often responsible for inflammatory processes (Manichanh et al., 2006; Vanderploeg et al., 2010). In the case of Irritable Bowel Syndrome (IBS), which is the most common functional gastrointestinal disorder in western countries, alterations in the gut microbiota have also been detected (Vanner, 2008; Yamini and Pimentel, 2010; Durban et al., 2012). Although no consensus has been reached regarding the association between specific bacteria and IBS, the gut microbiota of IBS patients has a reduced diversity. Moreover, IBS often follows bouts of gastrointestinal infection and there is evidence to suggest that antibiotics may play a role in the pathogenesis of the disorder (Mendall and Kumar, 1998).

### Deregulated Metabolism

Increasingly, the gut microbiota is being established as an important factor in the regulation of host metabolism, in particular as it relates to energy homeostasis and adiposity. Several metabolic disorders have been linked with gut microbiota dysbiosis. In particular, obesity has been associated with phylum-level changes in the gut microbiota, reduced bacterial diversity, and altered representation of bacterial genes and metabolic pathways, differences that endow the obesity-associated microbiota with an increased capacity to harvest energy from the diet (Backhed et al., 2004; Turnbaugh et al., 2006, 2009). This is in line with the fact that long-term exposure to antibiotics is associated with increased body mass index, both in humans (Thuny et al., 2010; Ajslev et al., 2011; Angelakis et al., 2012) and in farm animals, where low-dose antibiotics have long been used to promote weight gain (Burch, 1996). Moreover, recent work in mice has shown that early antibiotic exposure can cause obesity even with normal dietary intake (Cho et al., 2012). Antibiotic use is therefore emerging as an important risk factor for the development of obesity. In addition, it may also contribute to the onset of metabolic syndrome in obese individuals. The metabolic syndrome is a cluster of metabolic conditions that increase the risk for cardiovascular disease, fatty liver disease, steatohepatitis and type 2 diabetes. The advancement from obesity to metabolic syndrome appears to involve the establishment of a state of chronic low-grade inflammation, which could be exacerbated by antibiotic use (Emanuela et al., 2012; Francino and Moya, 2013).

On the other hand, antibiotics have also recently been implicated in increasing the risk for type 1, insulin-dependent diabetes, an autoimmune disease whose incidence has been steadily going up in industrialized countries in the last decades. In an epidemiological study involving a large UK population, the repeated use of penicillin, cephalosporins, macrolides, or quinolones was associated with increase in diabetic risk (Boursi et al., 2015). In a mouse model of type I diabetes, different antibiotic treatments that altered gut microbiota composition were also shown to significantly increase the incidence of the disease (Candon et al., 2015).

## Through What Mechanisms Do Gut Microbiota Alterations Affect Immunity and Metabolism?

Besides their direct ecological effects on the composition of the gut microbiota, antibiotics affect the manner in which this community interacts with the host and regulates basic physiological processes. Regarding the immune system’s capacity to fight infections, antibiotics indirectly alter the effectiveness of both innate and adaptive immune responses. As microbiota composition changes, not only non-resistant organisms capable of outcompeting potential pathogens are lost, but the altered community will present a substantially different repertoire of microbial-associated molecular patterns (MAMPS) to the receptors located in immune and epithelial cells. This will result in an altered stimulation of receptors such as NOD1 and the Toll-like receptors (TLRs), which can cascade down through a variety of immune processes, including lymphoid tissue development, T cell differentiation, neutrophil priming, production of antibacterials, and cytokine release (Ubeda and Pamer, 2012). A series of experiments in mice have shown that antibiotic treatment can reduce the capacity to fight infections by Gram-positive organisms by decreasing the expression of bactericidal compounds and diminishing neutrophil-mediated killing (Brandl et al., 2008; Vaishnava et al., 2008; Clarke et al., 2010; Ubeda et al., 2010). In the case of the adaptive immune system, both the expression of Major Histocompatibility Complex genes in the small and large intestine and the levels of immunoglobulin G (IgG) in serum have been shown to decrease in reponse to amoxicillin-induced gut microbiota changes (Dufour et al., 2005).

On the other hand, the cellular and molecular mechanisms by which gut microbiota alterations impact immunotolerance have long been debated (Rautava et al., 2004; Romagnani, 2004; Penders et al., 2007a; Sjogren et al., 2009; Jutel and Akdis, 2011). The balance between the Th1 and Th2 helper cell subsets of the adaptive immune system was, until recently, thought to be the main condition required for maintaining immune homeostasis. In support of this notion, chronic inflammatory/autoimmune and allergic diseases are known to associate with excessive Th1 or Th2 activation, respectively (Abbas, 1996; Oboki et al., 2008). Nevertheless, important roles for Th17 cells and regulatory T cells (Tregs) have been demonstrated in diseases that had classically been defined as Th1 or Th2-mediated (Nakae et al., 2002; Murphy, 2003; Oboki et al., 2008; Akdis and Akdis, 2009). In the current view, it is a new cellular balance that is considered critical for immune homeostasis: that between the Tregs and their effector cells, the different Th subsets. Alterations of the gut microbiota disrupt this balance, resulting in the deregulation of immune responses that can promote a variety of disease outcomes (Wills-Karp et al., 2001; Yazdanbakhsh et al., 2002; Rautava et al., 2004; Rook and Brunet, 2005; Penders et al., 2007b; Shen et al., 2014).

The generation of Tregs has indeed been shown to depend on the crosstalk between the gut microbiota and the immune system (Strauch et al., 2005). Experiments in mice or in *in vitro* cell cultures have revealed different specific bacteria and bacterial products that are capable of Treg cell induction (Kline, 2007; Baba et al., 2008). For instance, *Bacteroides fragilis* (Round and Mazmanian, 2010) and *Clostridium* species belonging to phylogenetic groups IV and XIV (Atarashi et al., 2011) promote the differentiation of T cells into Tregs in mice. In contrast, the Segmented Filamentous Bacteria (SFB) rather promote the differentiation of pro-inflammatory Th17 cells (Ivanov et al., 2009). This highlights the basic concept of different microbes driving the differentiation of naïve T cells into different subtypes. In humans, however, SFB are not commonly encountered in the gut microbiota, and these bacteria probably do not play any relevant role.

The routes through which antibiotic-induced microbiota alterations disrupt the balance among T cells and disturb immune homeostasis are being investigated in experimental mice models. Vancomycin, which kills Gram-positive bacteria, has been shown to cause a reduction of the number of Tregs in the lamina propia of the colon and to impair the induction of Th17 cells (Atarashi et al., 2011). On the other hand, a cocktail of antibiotics administered to two-week-old mice resulted in a reduced expression of TLRs and cytokine profiles promoting a Th2 response (Dimmitt et al., 2010). Similarly, kanamycin administered to three-week-old mice resulted in reduction of Peyer’s patch cellularity and in immune responses skewed toward Th2. Importantly, subsequent colonization with different bacterial species had very different effects: *Enterococcus faecalis* and *Lactobacillus acidophilus* reversed or attenuated the changes, respectively, whereas *Bacteroides vulgatus* actually caused their exacerbation. This underscores again the very different roles that specific types of bacteria play in relation to immune balance (Sudo et al., 2002).

Antibiotic-induced dysbioses are also likely to influence numerous immune and metabolic outcomes through routes that affect the intestinal milieu’s overall inflammatory tone. In this regard, microbiota alterations can result in a decrease of IgA, a non-inflammatory immunoglobulin involved in pathogen and allergen exclusion (Rautava et al., 2004; Penders et al., 2007a; Cerutti and Rescigno, 2008). In addition, metronidazole has been shown to cause a decrease in the expression of Muc2, the major component of the mucin layer (Wlodarska et al., 2011); thinning of this layer would result in a more direct contact between gut microbiota and epithelium, with likely increases in innate immune stimulation and inflammation. Recent work in mice has demonstrated that antibiotics can promote inflammation by increasing translocation of native colonic bacteria across the intestinal epithelium. Such translocation requires the participation of both immune dendritic cells and colonic goblet cells, and translocation enhancement results from the decrease in microbial signals received by the goblet cells (Knoop et al., 2015).

Inflammation-enhancing alterations in the gut microbiota, such as can be produced by antibiotic exposure, are likely to play a predominant role in the case of metabolic disorders such as obesity, metabolic syndrome and diabetes. The microbiota has been shown to contribute to the chronic low-grade inflammation that is associated with an excess of adiposity and that likely promotes the progression from obesity toward the metabolic syndrome. In this respect, the microbiota alterations induced by High Fat Diets (HFD) involve an increase of bacteria containing lipopolysacharides (LPS) in the cell wall, resulting in higher serum levels of this pro-inflammatory molecule, and experiments mimicking the HFD state through continuous subcutaneous infusion of LPS result in the induction of some aspects of metabolic syndrome (Cani et al., 2007). A deficiency in TLR5 also results in microbiota alterations that induce metabolic syndrome conditions such as obesity, insulin resistance and dyslipidemia. Moreover, the dysbiotic microbiota in itself is capable of inducing these disorders, as they could be reproduced in experiments in which the microbiota of TLR5-deficient mice was transplanted into germ-free recipients (Vijay-Kumar et al., 2010). Inflammation was likely involved in the onset of the observed metabolic conditions, as the wild-type mice with the transplanted dysbiotic microbiota had higher intestinal levels of the pro-inflammatory molecules TNFα and IL-1β.

Another main route through which microbiota dysbioses will induce their effects on immunity and metabolism is likely to be the alteration of short-chain fatty acids (SCFA) production. Intestinal microbes consume non-digestible carbohydrates to produce SCFAs, particularly acetate, propionate, and butyrate, which are used locally by colonocytes or transported across the gut epithelium into the bloodstream. SCFAs are major players in the maintenance of gut physiology and integrity, promote immune and metabolic homeostasis and have important anti-inflammatory and antitumorigenic effects (Bindels et al., 2012; Tan et al., 2014). In particular, SCFAs interact with G-protein-coupled receptors (GPCRs) to regulate fat deposition (Samuel et al., 2008), and improve insulin secretion through modulation of the levels of the GLP1 hormone (Tolhurst et al., 2012). The specific composition of the microbiota alters the types and levels of SCFA that can be formed, impinging on numerous physiologic processes that are differentially affected by acetate, propionate, and butyrate (Macfarlane and Macfarlane, 2011). In addition to producing SCFAs, the gut microbiota is responsible for converting the primary bile acids synthesized in the human liver into secondary bile acids, also involved in promoting glucose homeostasis through GPCR binding (Thomas et al., 2009). Antibiotic-induced microbiota alterations have been shown to alter bile acid metabolism and insulin sensitivity in both humans and mice (Vrieze et al., 2014).

## The Gut Microbiota as Reservoir of Antibiotic Resistances

The dysbioses brought about by antibiotics bear the added disadvantage of enriching the microbiota in resistant organisms. The human gut microbiota has been established as a significant reservoir of antibiotic resistances (Salyers et al., 2004; Seville et al., 2009; Sommer et al., 2009; Ghosh et al., 2013; Moore et al., 2013; Pehrsson et al., 2013; Card et al., 2014; Fouhy et al., 2014a,b; Hu et al., 2014; Lu et al., 2014; von Wintersdorff et al., 2014; Clemente et al., 2015; Field and Hershberg, 2015). The scale of the problem can be gaged by the fact that an analysis of 252 fecal metagenomes from different countries identified resistance genes for 50 out of the 68 antibiotic classes and subclasses that were being screened for, with an average of 21 per sample (Forslund et al., 2013). This study, the largest population-level analysis of the intestinal resistome to date, also showed that the abundance of antibiotic resistance genes (ARGs) is highest for antibiotics that have been longer in the market and for those approved for animal use, such as tetracycline, bacitracin and the cephalosporins. Also, European samples showed enrichment in resistances to vancomycin in comparison to samples from the US, where an analog of vancomycin used to treat animals in Europe was never employed. Moreover, the abundance of ARGs was higher in samples from Southern Europe than in those from Northern Europe, and correlated with measures of total outpatient antibiotic use in the different countries. This suite of observations confirms the notion that a higher exposure to antibiotics increases the likelihood of resistance acquisition by the gut microbiota.

Alarmingly, not only the microbiota of adults constitutes a resistance reservoir, but children and infants also harbor a variety of ARGs (Gueimonde et al., 2006; Mitsou et al., 2010; de Vries et al., 2011; Zhang et al., 2011; Alicea-Serrano et al., 2013; Ghosh et al., 2013; Field and Hershberg, 2015; Moore et al., 2015). Recent analyses have shown that, in fact, numerous ARGs can already be identified in feces of 1-week-old babies and even in meconium, the first deposition of newborns, which is formed by material accumulated in the gastrointestinal tract during fetal life (Gosalbes et al., 2015). Remarkably, ARGs are detected not only in adults and children that have undergone antibiotic treatments, but also in infants (Zhang et al., 2011; Fouhy et al., 2014a; Field and Hershberg, 2015; Gosalbes et al., 2015) and in isolated human populations (Pallecchi et al., 2007; Clemente et al., 2015) that have never been administered antibiotics. This indicates that ARGs can be stably maintained in the human gut microbiome in the absence of direct antibiotic selection, and is consistent with the fact that ARGs can be detected in a large range of natural environments, including those expected to have little exposure to antibiotics derived from human usage (Field and Hershberg, 2015).

In the case of infants, resistances may be vertically inherited, as maternal gut microbes can be transmitted to the offspring (Vaishampayan et al., 2010), with several lines of evidence indicating that such transfer actually starts before birth (Jimenez et al., 2005, 2008; Steel et al., 2005; DiGiulio et al., 2008; Gosalbes et al., 2013, 2015; Aagaard et al., 2014). Several studies have demonstrated shared ARG pools between mother and infant fecal samples, and, in some cases, the presence of the shared ARGs in meconium, colostrum or breast milk (de Vries et al., 2011; Zhang et al., 2011; Gosalbes et al., 2015). Nevertheless, these studies have also detected ARGs in infants that were not present in the mothers and were most probably acquired from other sources. Regarding remote human populations (Pallecchi et al., 2007; Clemente et al., 2015), the presence of ARGs in their microbiotas suggests two possibilities: (i) either their ARGs are ancestral and were present before the rampant spread of resistance due to human antibiotic use, presumably due to selective pressures imposed by naturally occurring antibiotics, or (ii) they have been acquired recently by dispersion of antibiotic resistant strains from other areas and/or by horizontal transfer of genes from such strains to their local bacterial populations. Phylogenetic and population genetic analyses should be able to discern between these alternatives.

Importantly, the human gut, given its enormous density of bacterial cells and species richness, is likely to be especially prone to horizontal gene exchange and to contribute to the spread and reassortment of ARGs among bacterial taxa. Transfer of ARGs between gut microbiota isolates of the genus *Bacteroides*, as well as between *Bacteroides* and Gram-positive bacteria, has been documented (Shoemaker et al., 2001). Identical ARG sequences have actually been identified in bacteria coexisting in the gut of a single individual, including different strains of *E. coli* (Karami et al., 2007) as well as distantly related organisms (de Vries et al., 2011). Experimental work has confirmed that ARG-carrying transposons can be transferred between bacterial species in the guts of rats and mice (Doucet-Populaire et al., 1991; Alpert et al., 2003; Bahl et al., 2004). Furthermore, the transfer of conjugative transposons can be stimulated 100- to 1000-fold by low concentrations of antibiotic (Whittle et al., 2002). Of most concern, the ARGs present in the gut microbiota can also be horizontally transferred from and to incoming pathogenic species, as indicated by the fact that many of the resistance genes identified in human gut isolates are identical at the nucleotide level to resistance genes from human pathogenic isolates (Sommer et al., 2009). Therefore, the human gut can be considered, not only a site of accumulation of ARGs, but also an environment where these genes can spread across species boundaries.

## Summary and Outlook

**FIGURE 1:**
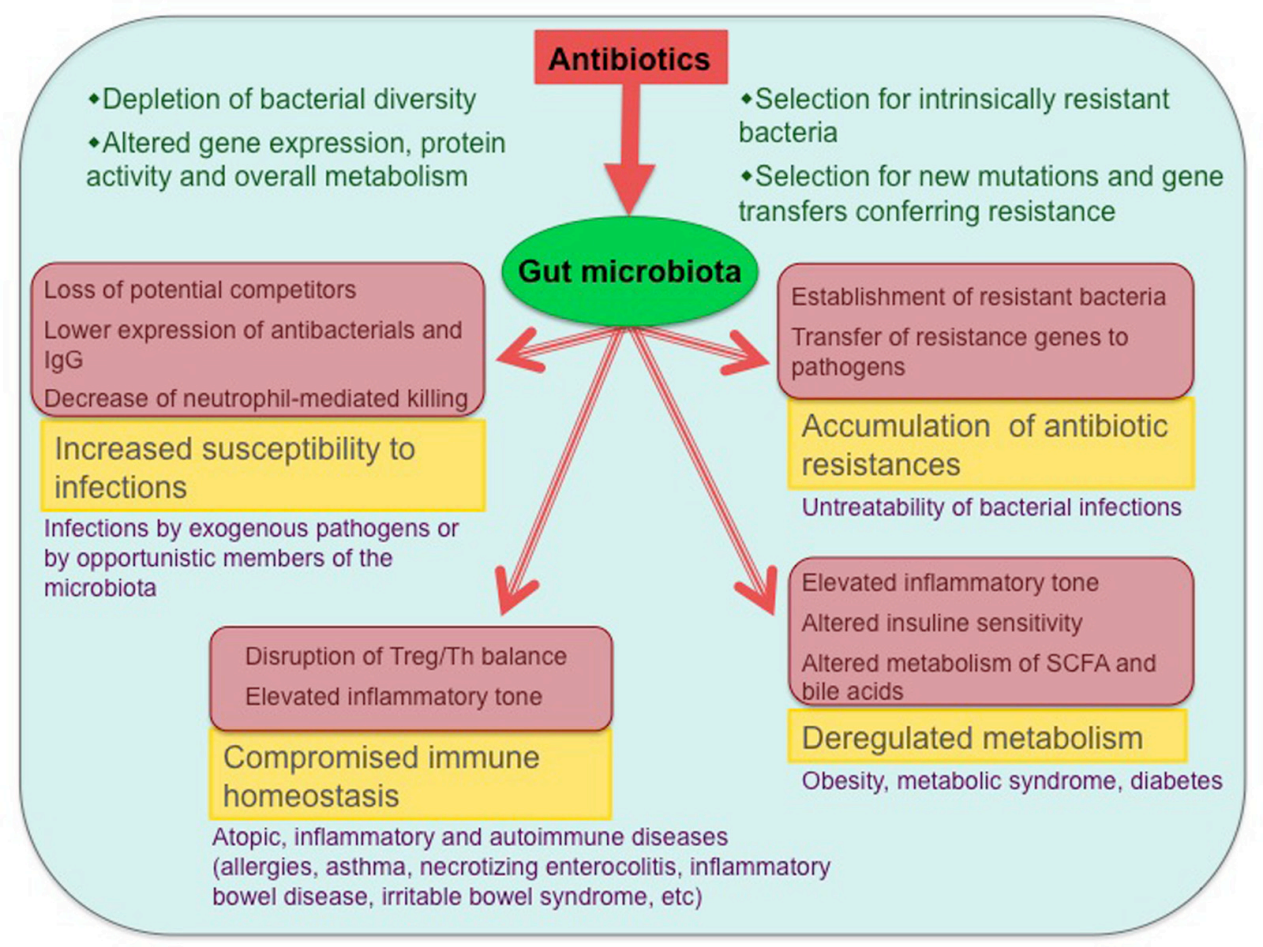
**Antibiotic effects on the gut microbiota and associated health problems.** The main biological consequences of antibiotic-induced dysbioses and the potential diseases that can ensue from them are shown (only diseases with published evidence of association with antibiotic exposure are included). Involved mechanisms are shown in pink-shaded boxes.

It is clear that the excessively widespread use of antibiotics has created many threats. These include the increasing resistance of bacterial pathogens to antibiotics, which has become a global challenge for infection control. But the effects of excessive antibiotic exposure can be seen, not only in pathogenic bacteria, but also in the symbiotic microbiotas of the human body (Francino and Moya, 2013). As a result, the microbiota imbalances caused by antibiotics can negatively affect health in numerous manners and for long periods of time. The range of problems potentially generated by antibiotic-induced microbiota dysbioses, as reviewed in this paper, is summarized in **Figure 1**. In light of this knowledge, and given that bacterial infections remain a major public health concern, strategies are needed to minimize the negative consequences of antibiotics when their administration is required. Use of probiotic bacteria aimed at impeding dysbiosis or at reestablishing the gut microbiota after antibiotic treatment is a promising approach. On the other hand, strategies could also be aimed at reestablishing the interactions altered by antibiotic treatment through the targeted use of bacterial molecules that bind specific innate immune receptors (Ubeda and Pamer, 2012). Much further research is needed to delineate the best manners in which bacteria and bacterial products can be employed to counteract the deleterious effects of antibiotics on the gut microbiota and its multiple interactions with immunity and metabolism.

An equally important line of research should aim at understanding the patterns of dispersion and expansion of antibiotic resistant strains in the human gut microbiome, as well as the routes of gene exchange that may distribute resistances across different gut taxa. Virome and mobilome analyses should enable us to establish the associations of ARGs with specific genetic elements, providing clues to the paths through which they can be disseminated within and across gut microbial communities. Understanding the flow of resistances within the gut microbiome will contribute an important piece to the puzzle of antibiotic resistance epidemiology, which needs to integrate information from human and environmental microbiomes to the analysis of resistance spread in pathogenic isolates.

## Conflict of Interest Statement

The author declares that the research was conducted in the absence of any commercial or financial relationships that could be construed as a potential conflict of interest.
